# Novel non-invasive technology for cardiac output determination

**DOI:** 10.1186/cc13331

**Published:** 2014-03-17

**Authors:** U Borg, D Iyer, Q Huang, Y Li, C Baker

**Affiliations:** 1 Covidien, Longmont, CO, USA

## Introduction

The aim of this animal study was to determine the feasibility and accuracy of a new non-invasive system for determination of cardiac output (CO) compared with a known device (PiCCO; Pulsion Medical). In ICUs and ORs, hemodynamic management using goal- directed therapy has been shown to improve patient outcomes [1],[2]. The invasiveness of current technology may prevent clinicians from obtaining hemodynamic information. We developed a non- invasive system for detection of hemodynamic variables utilizing a photo-acoustic sensor (PA-S) and indicator dilution. The PA-S utilizes a combination of a miniature ultrasound sensor, a laser diode and an optical detector to accomplish the measurement.

## Methods

Five pigs weighing 25 to 29 kg were used in the study approved by the local animal use and care committee. The animals were anesthetized, medically paralyzed, intubated and mechanically ventilated. Central venous and arterial catheters were placed for injection of the indicator and connection to the PiCCO system. The PA-S was placed in contact with the skin over the saphenous artery on the animal and adjusted to receive the maximum acoustic signal. The adjustment included the depth of ultrasonic penetration and the exact location on top of the blood vessel. Each indicator dilution was accomplished using three central venous injections of 15 ml normal saline at room temperature. Variation in CO was accomplished by removing up to 50% of the blood volume after splenectomy. The PA-S results were obtained using the empirical regression method. The resulting cardiac output readings from the PA-S were compared with the PiCCO readings.

## Results

The correlation between the PA-S and the PiCCO system was (*R*^2 ^= 0.746) over a range of CO from 1 to 6 l/minute. The Bland-Altman analysis demonstrated a bias of -0.05 l/minute and precision of 0.50 l/ minute.

## Conclusion

This animal study demonstrated the feasibility of the new PA-S for determination of indicator dilution CO in a limited range. The system showed good correlation with the PiCCO system even in the case of vasoconstriction as a result of blood loss. Since the PA-S utilizes transpulmonary indicator dilution, other variables such as intrathoracic blood volume, global end-diastolic volume and extravascular lung water can be calculated. Additionally, continuous CO can be obtained from the optical sensor signal utilizing arterial waveform analysis.
